# *Sargassum latifolium*-mediated Se/CuO/MgO/ZnO nanocomposite enhances salt tolerance in *Phaseolus vulgaris* and exhibits antibacterial and antioxidant activities

**DOI:** 10.1038/s41598-026-59437-3

**Published:** 2026-07-03

**Authors:** Sultan M. Alsharif, Ahmed M. Eid, Ahmed M. Alemam, Mohamed A. Amin, Faisal A. Alraddadi, Moayad S. Waznah, Saad El-Din Hassan, Abeer Muhammad Almutrafy, Fatmah O. Sefrji, Abeer S. Albalawi, Mohamed Ali Abdel-Rahman, Mohamed E. Elhady, Amr Fouda

**Affiliations:** 1 Department of Biology, College of Science, Taibah University, 42353 Madinah, Saudi Arabia; 2 Botany and Microbiology Department, Faculty of Science, Al-Azhar University, Nasr City, 11884 Cairo Egypt; 3 Biology Department, Faculty of Science, Al Baha University, Al Aqiq, Saudi Arabia

**Keywords:** Tetrametallic nanocomposite, *Sargassum latifolium*, Abiotic stress, Common bean, Multi-drug-resistant, DPPH, Biochemistry, Biotechnology, Microbiology, Plant sciences

## Abstract

Macroalgae represents a powerful, renewable bioplatform for generating high-performance nanomaterials that bridge sustainability with advanced biotechnological applications. However, crop production and multi-drug resistance microbes are considered the main challenge worldwide. Also, the incorporation of macroalgae metabolites to generate multimetallic nanocomposite, as a new active compound, remains unexplored. In this work, the brown macroalga *Sargassum latifolium* was exploited as a robust biofactory for the green fabrication of multifunctional active tetrametallic Se/CuO/MgO/ZnO (TSCMZ) nanocomposite. The biosynthesized TSCMZ were characterized by FT-IR which explains the role of different algal-active metabolites in biofabrication process. Also, TEM, SAED, and EDX confirm the spherical shape with average sizes of 24  *nm* and the presence of metallic elements as main nanocomposite component. Polycrystalline architecture of synthesized nanocomposite was confirmed by XRD analysis. Under field conditions, *Phaseolus vulgaris* L. exposed to 100 mM NaCl experienced drastic impairments in growth, metabolism, and yield stability. Remarkably, foliar application of TSCMZ (50–200 ppm), especially at 200 ppm, significantly mitigate salt stress, elevating metabolic performance, and recovering key yield attributes. This enhancement was accompanied by substantial increases in chlorophylls (a, b, and a + b), carotenoids, free proline, carbohydrate and protein biosynthesis, indicating strengthened osmotic adjustment and redox homeostasis. Beyond its agronomic impact, TSCMZ displayed striking antibacterial potency, with low MIC values (12.5–25 µg mL^–1^) against multidrug-resistant (MDR) pathogens including *E. coli* and *Klebsiella pneumoniae*. Moreover, the algal-mediated tetrametallic nanocomposite demonstrated high antioxidant strength through potent DPPH radical scavenging at ≥ 125 µg mL^–1^. Collectively, these findings demonstrate that TSCMZ is a salt-stress mitigation treatment and have the efficacy to inhibits the growth of MDR bacterial strains.

## Introduction

Macroalgae, also names as seaweeds, are macroscopic and multicellular marine autotrophs and its length reached in some species to several meters. Macroalgae are taxonomically into four groups: red species (phylum Rhodophyta), green species (phylum Chlorophyta), brown species (phylum Ochrophyta), and blue-green ones (phylum Cyanobacteria)^1^. Recently, macroalgae are involved as a sustainable solution for different environmental challenges such as phycoremediation, valorization of lignocellulosic materials, carbon scavenging, and important products production.

Salinity is the most severe abiotic stress causing worldwide decreasing crop production in regions characterized by water scarce. Plant growth is inhibited due to salinity stress causing impair physiological processes, reducing growth, ultimately significant yield losses, and hence global food protection highly affected. Consequently, considerable scientific attention has been directed toward developing innovative and sustainable strategies capable of enhancing crop tolerance to salinity and other abiotic stresses^2^.

The utilization of live algal biomass or drying (non-viable) as an eco-friendly biological platform for the rapid and efficient biosynthesis of nanoparticles is increasingly being adopted. Macroalgae possess several advantageous features, including high biomass productivity, rapid growth rates, high adsorbs, and reduced metal ions. The cultivation of microalgae and/or macroalgae can be achieved in absence of chemical fertilizers or synthetic additives. In contrast to other biological resources, algae can be harvested multiple times throughout the year^3^. *Sargassum* spp. are brown macroalgae that are distributed along the Red Sea coast of Egypt as well as across tropical and subtropical regions worldwide. These algal species are rich in diverse bioactive phytochemicals with well-documented antimicrobial, anticancer, anti-hypercholesterolemic, anti-inflammatory, hypolipidemic, antineoplastic, antiapoptotic, and antioxidant properties. Such compounds include polysaccharides, pigments, terpenoids, vitamins, sargaquinoic acids, carrageenan, steroids, carbohydrates, sargaquinols, macro- and microelements, and glycerides^4^. Fucoxanthin, tocopherol, and β-carotene are the most pigments found in brown algae and exhibit anti-obesity, anticancer activities, and effectively scavenge reactive oxygen species^5^. Recently, biogenic nanotechnology has gained attention as a sustainable approach instead of physical and chemical methods which expensive, energy-intensive, and involve hazardous substances^6^. Biological synthesis routes are particularly important in bioengineering, as nanoparticles generated by plants or microorganisms are environmentally benign and sustainable^7,8^. Among biological synthesis is utilizing macroalgal metabolites to fabricate nanoparticles and/or nanocomposites for various biotechnological applications.

Recent advances in nanotechnology have demonstrated that nanoparticles can improve plant growth and resilience of various stresses under environmental field conditions. Salinity-affected soil currently accounts for nearly 20% of irrigated agricultural lands globally, and projections indicate that this proportion may exceed 50% by 2050 if appropriate mitigation strategies are not implemented^9^. Among leguminous crops, *Phaseolus vulgaris* L. (common bean) is of particular economic importance due to its high nutritional value and widespread cultivation. However, common bean are highly salinity stress sensitive, which markedly restricts their growing and yield potential, highlighting the urgent need for effective approaches to enhance their tolerance under saline conditions^10^.

Antibiotic resistance has emerged as a foremost health concern global. Once primarily associated with hospital-acquired infections, it has now become widespread due to inappropriate antibiotic usage, overprescription of broad-spectrum drugs, limited availability of targeted therapies, and the rapid development of resistant strains, facilitating the dissemination of pathogens that are unresponsive to conventional antibiotics. It is projected that by 2050, effective antibiotics for treating such infections may no longer be available^11^. Moreover, the prevalence of multidrug-resistant bacteria contributes to increased mortality rates among cancer patients, as treatment options are limited and knowledge regarding bacterial infections in individuals with solid tumors remains insufficient^12^.

Accordingly, innovative strategies are being actively investigated to combat multidrug resistance and to identify precise and efficient therapeutic approaches for cancer treatment. Nanomedicine offers promising solutions for fundamental limitations related to conventional cancer diagnosis and therapy, while also providing effective treatments for bacterial infections that are difficult to eradicate. In addition, nanomedicine may help circumvent existing mechanisms responsible for the development of antibiotic resistance^8^. Nanomedicine represents a rapidly advancing discipline that employs nanobiotechnology tools, such as nanoparticles (NPs) and nanodrugs, for disease surveillance, diagnosis, treatment, and prevention. By exploiting advanced nanoscale technologies, this emerging field holds significant potential for managing diseases that are currently considered incurable^13^. Nanomaterials, typically defined by dimensions below 100 *nm*, show unique properties compared to bulk materials. These nanomaterials have found extensive applications in agriculture, medicine, and environment.

Within this framework, the present study aimed to explore the algal-mediated green fabrication of a tetrametallic Se/CuO/MgO/ZnO (TSCMZ) nanocomposite using an aqueous extract of *Sargassum latifolium*. The biosynthesized nanocomposite was extensively characterized through advanced physicochemical analyses, including FT-IR, TEM, SAED, XRD, and EDX. In addition, the effectiveness of the TSCMZ nanocomposite in mitigating salinity-induced stress in *Phaseolus vulgaris* under field conditions was assessed, along with its antibacterial activity against multidrug-resistant bacteria and its antioxidant potential. The novelty of the current investigation is the first report utilizing *Sargassum latifolium* aqueous extract for green synthesis of tetrametallic nanocomposite containing for metals (Se/CuO/MgO/ZnO) and integrated it in agricultural and medical sector. In addition to antibacterial activity of four selected metals, it was selected for formation of tetrametallic nanocomposite for alleviating salt stress because of Se has antioxidant activity which upregulate stress enzymes, such as thioredoxin reductase and glutathione peroxidase, leads to decreasing lipid peroxidation and hence protect membrane integrity under stresses. Also, Cu and Zn are required as cofactors for superoxide dismutase and cytochrome c-oxidase which enhance electron transport during respiration and photosynthesis. Moreover, Mg is the main element of chlorophyll and activator for ATPase enzyme leads to protecting chlorophyll from degradation under stresses. On the other words, salinity stress causing deleterious effects to photosynthetic mechanism, oxidative stress, and ionic imbalance in plants. There is no single metal that can address these negative effects. Therefore, the combination between these four elements can improve tolerance of plants to salt stress and alleviate its negative effects.

## Materials and methods

### Macroalgae strain

The brown macroalga *Sargassum latifolium* (Turner) was harvested in June 2023 from the Red Sea shoreline at Hurghada, Egypt after taken an appropriate permission from Red Sea shoreline organizing committee with number RS-6-2023-112. The algal material collection was conducted in accordance with the local and national regulations. Immediately following collection, the algal samples were transferred to the Phycology Lab., Faculty of Science, Al-Azhar University for taxonomic identification (by Dr. Ehab El-Belely, Associate Professor of Applied Mycology) using standard morphological keys^14^. After identification, a voucher specimen was deposited in the Lab., with accession number of M.A.A-2023-65. Prior to subsequent processing, the collected materials were thoroughly rinsed with deionized water to eliminate residual salts and surface debris.

### Tetrametallic Se/CuO/MgO/ZnO (TSCMZ) nanocomposite fabrication

#### Algal extract preparation

After collecting freshly algal biomass, it was rinsed thrice with tap-water to remove any sand, epiphytes, and other debris. Afterword, the cleaned biomass was air-dried at 30 °C for 4–7 days till weight was constant. Using stainless steel blender (Philips, 500 series, HR2767, China), the dried algal biomass was mechanically powdered, which pass thoroughly stainless-steel sieve to collect the uniform fine powder.

A 10 g of fine powder was mixed with deionized water, 100 mL, and heated in a digital stainless-steel water bath (Precision SWB-15, Thermo-Fisher, China) set at 60 °C with stirring (200* rpm*) for 1 h. After that, the mixture was cooled at 30 °C and subjected to centrifugation at 10,000 *rpm* for 10 min to collect the clear supernatant (clear from particulate and attached matter), which is used forward to nanocomposite synthesis^15^.

#### Tetrametallic nanocomposite fabrication

For nanocomposite fabrication, aqueous solutions of Na_2_SeO_3_ (selenium source, 98.0%, Sigma-Aldrich, China), Cu(CH_3_COO)_2_⋅2H_2_O (copper oxide source, 99.0%, Sigma-Aldrich, China), Mg(NO_3_)_2_⋅6H_2_O (magnesium oxide source, 99.0%, Merck, Germany), and Zn(CH_3_COO)_2_⋅2H_2_O (zinc oxide source, 99.0%, Fisher-Scientific, China) were freshly prepared. The stock solution from each metal was prepared with a concentration of 30 mM followed by withdraw 10 mL from each stock solution and mixed to form totally 40 mL which added to 60 mL of algal extract, giving a final volume of 100 mL. At the end, each metal concentration in the final volume is 3 mM. This concentration was selected based on preliminary investigation (not published) where 3 mM is the optimum concentration to spray on *Phaseolus vulgaris* L. without any disease symptoms. In preliminary phytotoxicity experiments on *P. vulgaris* plant, higher concentration above 3 mM causes leaf damage and nanocomposite aggregate on the leaf surface, whereas lower concentration < 3 mM did not show significant salinity alleviation.

**Fig. 1:**
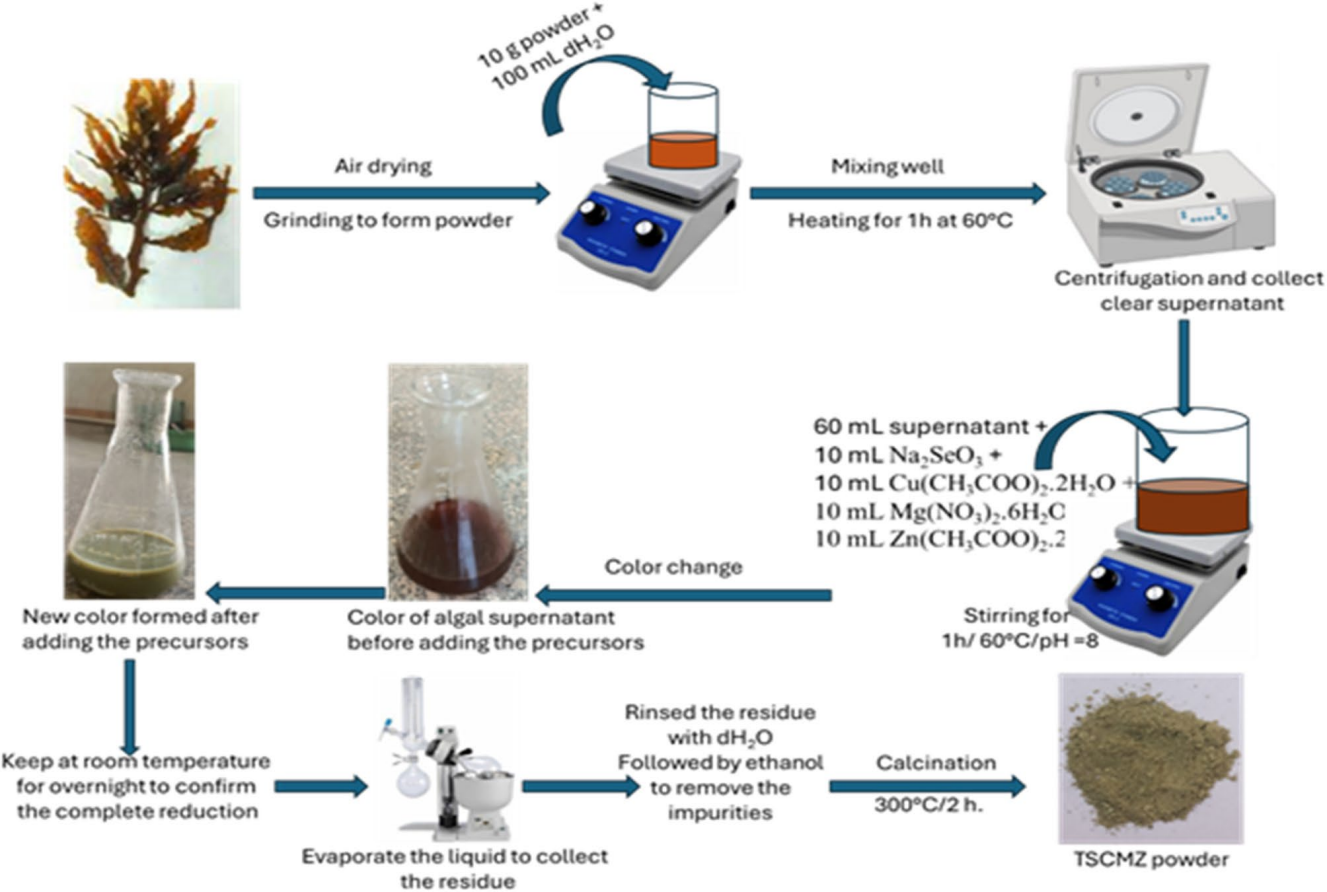
Schematic description of the steps involved green fabrication of the TSCMZ nanocomposite utilizing *S. latifolium* aqueous extract.

The reaction mixture was adjusted to pH 8.0 using 0.1 M NaOH with stirring at 60 °C in magnetic stirring water bath (Precision SWB-15, Thermo-Fisher, China) until the color changed from reddish-brown to greenish brown (Fig. 1), indicating initial nanoparticle formation. Subsequently, the mixture was kept overnight at 35 °C to ensure complete reduction and stabilization mediated by algal metabolites.

The obtained suspension (100 mL) was concentrated to 20 mL using a rotary evaporator (50 °C, 100 *rpm*, 100 mbar pressure, for 30 min). The concentrated residue (20 mL) mixed with 10 mL of deionized H_2_O followed by centrifugation for 10,000 *rpm* for 10 min to collect the pellets. This step was repeated three times to remove salt and water-soluble unreacted substances. After that, the collected pellet was washed thrice with 10 mL ethanol and subjected to centrifugation after each washing to remove organic impurities and algal remaining residue. The purified pellet was dried at the first at 70 °C for 5 h., followed by calcination at 300 °C for 2 h to yield the final dried TSCMZ nanocomposite powder (Fig. 1), which was preserved for further characterization and analyses.

### Characterization of TSCMZ nanocomposite

Cary-630- Fourier transform infrared (FT-IR, Tokyo, Japan) spectroscopy was used for detecting functional groups involved in nanocomposite formation over ranges 400 cm^−1^ to 4000 cm^−1^ wavenumbers. The FT-IR analysis was achieved separately on two samples, the crude algal extract and purified nanocomposite powder. Each sample (30 mg of TSCMZ nanocomposite or 50 µL of crude algal extract) was thoroughly mixed with KBr, blending well, followed by pressed under vacuum drying to prepare a disk before scanning^16^. The morphological features of the nanocomposite, including particle size, shape, and aggregation state, were analyzed by JEOL-1010-transmission electron microscopy (Tokyo, Japan). A few drops of the dispersed powder solution (10 mg), previously sonicated in high-purity Milli-Q H_2_O for 10 min to ensure uniform dispersion, were deposited onto carbon-copper TEM grids. Using blotting paper, the excess liquid on TEM grid was removed before being examined at 100 kV^17^. During TEM analysis, the crystalline of fabricated nanocomposite was detected by SAED (selected area electron diffraction) patterns. The crystalline structure confirmed by X-ray diffraction (XRD, PANalytical X’Pert-Pro-MRD). The analysis was performed with radiation source of CuKα (λ = 1.54 Å) at 40 kV and 30 mA. The analysis was achieved over 2θ values of 5° to 80°, revealing characteristic Bragg reflections corresponding to the different metals and metal oxides present in the TSCMZ nanocomposite. The mean crystallite nanocomposite size was estimated by the equation of Debye–Scherrer, as shown below^18^:

1 $$Average crystallite size= \frac{0.94 \times 1.54}{\beta cos\theta }$$

where 0.94 and 1.54 are the Scherrer constant and X-ray wavelength respectively, β and θ corresponds to the full width at half maximum and the Bragg diffraction angle respectively. Elemental composition of the biogenic tetrametallic nanostructure was determined using JEOL-JSM6360LA-energy-dispersive X-ray (EDX, Japan) at 5 kV accelerating voltage.

### Field experiment design

#### Plant material and growth conditions

The common bean (*Phaseolus vulgaris* L.) seeds used in this study were obtained from the Agricultural Research Center (ARC), Ministry of Agriculture, Giza, Egypt. The field experiment was conducted in the Botanical Garden, Faculty of Science, Al-Azhar University, Department of Botany and Microbiology. The experimental work was carried out with official permission from the garden administration (Permission No. 102/24). No plant material was collected from private farmlands.

The surface of common bean (*Phaseolus vulgaris* L.) seeds was sterilized using sodium hypochlorite (0.1%) for 15 min, followed by rising with sterile H_2_O, and sown under field conditions following a completely randomized experimental design at Faculty of Science-Botanical Garden, Al-Azhar university, Egypt. Two salinity levels (0 and 100 mM NaCl) were used as treatments in combination with four concentrations of the TSCMZ nanocomposite (0, 50, 100, and 200 ppm), with three replicates for each treatment. The selection of salt concentration (100 mM) was achieved based on preliminary experiments for morphological parameters of *Phaseolus vulgaris* growing under different concentrations of NaCl (Table 1)*.* Plants were cultivated in plastic bags containing sandy loam soil (6 kg/each bag), where six seeds were initially planted per bag and later thinned to four uniform seedlings. The control plants were irrigated by fresh water whereas salt stress plants were irrigated by saline water (100 mM NaCl). The irrigation was carried out every 4 days with 500 mL for each pot for 4 weeks and increased the irrigation intervals to be every 7 days until the end of experiment. The salt stress induction mode was root-zone application, and the stress started after 15 days of sowing. The plant grown was achieved under natural field conditions without artificial photoperiod and the length of day during the experiment ranging from 11–12 h. Three fertilizations were done during the experimental period at 14, 28, and 35 days after planting. On the two first fertilizations, each pot awarded 240 mg dm^−3^ of MgNO_3_ and 250 mg dm^−3^ of KNO_3_. On the last fertilization, 100 mg dm^−3^ of urea was applied per pot. All pots (control, salinity only, salinity + nanocomposite) received identical amounts of fertilizations. Therefore, the differences between different treatments originated from the foliar nanocomposite, not differential nutrient supply. The Foliar spray was used for TSCMZ treatment as follows: 1.0 g of synthesized nanocomposite was suspended in 500 mL of distilled H_2_O containing Tween-20 (0.01% *v/v*) as surfactant. The previous suspension was sonicated using ultrasonic bath at 25°C for 30 min to ensure uniform suspension. *P. vulgaris* plant was subjected to foliar spray of the aerial portions by formed suspension with 50 mL/plant. The foliar spraying was made when the plants were 35 days following seeding. Morphological and biochemical assessments were performed 45 days after sowing, whereas yield parameters were recorded after 86 days of cultivation (growing season end).

**Table 1:** Preliminary experiment showing the effects of different concentrations of salinity on morphological parameters of *Phaseolus vulgaris* plant.

Treatments		Shoot lengths (cm)	Root lengths (cm)	Fresh weight of shoot (g)	Dry weight of shoot (g)
Salinity	0	19.73 ± 0.25a	6.32 ± 1.01a	0.79 ± 0.07a	0.042 ± 0.021a
	50 mM	17.67 ± 2.12b	5.57 ± 0.82b	0.56 ± 0.02b	0.03 ± 0.017b
	100 mM	13.63 ± 0.50c	4.76 ± 0.89c	0.21 ± 0.01b	0.02 ± 0.010c
	150 mM	5.13 ± 0.30d	2.12 ± 0.09d	0.09 ± 0.02c	0.01 ± 0.010d
	200 mM	4.61 ± 1.35d	1.83 ± 0.16e	0.05 ± 0.06cd	0.01 ± 0.012d

Different letters in the same column indicate significant differences (*P* ≤ 0.05).

#### Measurements of growth and yield parameters

The following parameters, shoot and root length and weights of fresh and dry biomass, were measured as vegetative growth parameter after 45 days of sowing. Whereas yield attributes such as the number of pods and seeds per plant, seed weight/plant, and weight of 100-seed were determined after 86-days.

#### Biochemical and physiological analyses

##### Photosynthetic pigment determination

Chlorophyll and carotenoid contents were quantified by grinding fresh leaf tissue (1.0 g) in 80% acetone (100 mL), followed by extraction and filtration. The absorbance of the clarified extract was measured at 470 nm, 649 *nm*, and 665 *nm*. Concentrations of photosynthetic pigments (chlorophyll-a (*Chl-a*), chlorophyll-b (*Chl*-*b*), total chlorophyll (*Chl a* + *b*), and carotenoids) were measured using the following equations^19^:

2 $$chl-a \left(mg per g plant tissue\right)={11.63}_{(A665)}-{2.39}_{(A649)}$$

3 $$chl-b \left(mg per g plant tissue\right)={20.11}_{(A649)}-{5.18}_{(A665)}$$

4 $$Total chl a+b \left(mg per g plant tissue\right)={6.45}_{(A665)}+{17.72}_{(A649)}$$

5 $$carotenoid \left(mg per g plant tissue\right)=\frac{1000 A470-1.82 chla-85.02chlb}{198}$$

where A is absorbance.

##### Plant metabolic content determination

Content of total carbohydrate was estimated by anthrone–sulfuric acid^20^, while concentration of protein was detected spectrophotometrically by Lowry method^21^.

##### Free proline determination

Content of proline was assessed by homogenizing dried plant tissue (0.5 g) in 3% sulfosalicylic acid (10 mL) and filtrate, which interact with ninhydrin and glacial acetic acid for 1 h under heating water bath, following by cooling, and extracted with toluene. Absorbance of final extract was detected spectrophotometrically at 520 *nm* (UNICO Vis Model 1200, USA)^22^.

##### Lipid peroxidation assessment

Malondialdehyde (MDA) content was used as an indicator for lipid peroxidation. In this assay, 0.2 g of fresh leaf tissue was homogenized in 5% trichloroacetic acid followed by centrifugation to obtain a clear supernatant, which was then reacted with 0.67% thiobarbituric acid under boiling conditions for 30 min. At different wavelengths (450, 532, and 600 *nm*), the absorbance of final product was measured, and calculate the MDA concentration using the following equation:

6 $$MDA concentration=6.45 \times \left(A532-A600\right)-0.56 \times A450$$

Results are represented as µmol MDA/g of fresh weight.

##### Antioxidant enzymatic activities

For this assay, 10 mL phosphate buffer (0.1 M, pH 6.8) was used to homogenize young leaves and terminal buds before being subjected to cooling centrifugation (20,000 *rpm*, 20 min, 2 °C). The activity of catalase (CAT), Peroxidase (POX), and Polyphenol oxidase (PPO) was measured using resulting supernatant^23,24^ as follows:

**CAT activity test:** 50 µL of enzyme extract interact with H_2_O_2_ (15 mM) in the presence of potassium phosphate buffer (100 mM, pH 7) for 1 min at 25 °C. The absorbance of final color was measured at 240 *nm*. CAT activity was defined as the quantity of enzyme necessary to decompose 50% of H_2_O_2_.

**POX activity test:** it is measured using the pyrogallol method, with absorbance recorded at 470 *nm*.

**PPO activity test** was assessed by monitoring absorbance at 395 *nm* following reaction with catechol in acetate buffer (pH 5.0) for 60 min at 30 °C. Enzyme activities were represented as changes in optical density/minute/g of fresh weight.

### Antibacterial activity

Five MDR bacterial isolates, *Escherichia coli*-ESBL1, *E. coli*-ESBL2, *E. coli*-ESBL3, *Klebsiella pneumoniae*-ESBL, and *K. pneumoniae*-XDR, were collected from Microbial Physiology Lab., Faculty of Science, Al-Azhar University. These strains were identified as extended-spectrum β-lactamase (ESBL) and extensively drug-resistant (XDR).

The antibacterial susceptibility of MDR isolates to phyco-derived TSCMZ nanocomposite was evaluated using the agar well diffusion method. Muller Hinton agar plates were streaked with 50 µL of bacterial suspension (5 × 10^5^ CFU/mL) before forming four wells (6 mm) in each plate and filled with 100 µL of suspended nanocomposite. The following nanocomposite concentrations, 6.25, 12.5, 25, 50, 100, 200, and 400 µg mL^–1^, were used compared to DMSO as solvent system (negative control) and gentamicin as positive control. For diffusion, the previous plates were kept in refrigerator for 1.0 h before being transferred to incubator for 24 h at 35 ± 2 °C. The results were recorded as diameter (mm) of inhibition zone (IZ) around each well. The lowest concentration forming IZ was identified as the MIC (Minimum inhibitory concentration) value^25^.

### Antioxidant activity

The scavenging of free radical by TSCMZ nanocomposite was evaluated using the DPPH assay. The nanocomposite was prepared in ethanol at two-fold serial dilutions from 1000 to 1.95 µg mL^–1^. A 1.0 mL of DPPH solution (prepared in ethanol with concentration of 0.1 mM) was mixed with 3 mL of nanocomposite solution, following incubation at 37 °C in dark condition for 30 min. Ascorbic acid was used as a positive control under identical experimental conditions. Following incubation, absorbance was measured at 517 *nm*, and calculate the scavenging radical percentages using the equation below^26^:

7 $$DPPH scavenging \%=\frac{{A}_{0}-{A}_{1}}{{A}_{0}}x100$$

where, A_0_ and A_1_ represent the optical density (OD) of the control and treated samples, respectively.

### Statistical analysis

Data were expressed as mean ± standard deviation (SD) of three independent experiments. Statistical differences among experimental groups were evaluated using one-way analysis of variance (ANOVA), where each strain–concentration combination was treated as an independent group, followed by Tukey’s post-hoc multiple comparison test using a significance level of *P* < 0.05.

## Results and discussion

### Green synthesis of tetrametallic nanocomposite using algal extract

Recently, algae exhibited high efficacy to accumulate different metals or its oxide and reduced it forming nanoscale structure. The fabrication of these nanostructures can be achieved *via *harnessing algal metabolites of micro or macroalgae. The biosynthesis of nanomaterials using macroalgae are promising model compared to biosynthesis of microalgae due to its high activity to metal ions hyperaccumulated, remodeling metals to different shapes and sizes, cost-effective, shorter generation times, and high metabolic production, which are used as reducing agents^27^. Macroalgae is characterized by wide range of metabolites, such as fatty acids, vitamins, oil, carbohydrates, bioactive substance (polyphenols), chlorophylls, pigments (such as xanthophyll or carotene), and others. The secretion and concentrations of these metabolites are varied based on species and used mainly as reducing of metals to nanostructure following by capping and increase the product stability^27,28^. The presence of these diverse metabolites not only making algal-mediated synthesis are promising candidates for green synthesis overing traditional (chemical or physical) methods, also impair unique features on the nanostructure which increase its applicability in different sectors^29^. For instance, bimetallic Ag/Fe-NPs was fabricated by harnessing metabolites of marine macroalgae *Galaxaura rugosa*^30^. The authors reported that the algal metabolites*,* including polyphenols, polysaccharides, and proteins, were utilized as reducing agent for metal ions to produce bimetallic nanostructure followed by capping the final product to improve its stability. Although there are different published investigations about the utilization of macroalgae for producing metallic or metallic oxides nanostructure, this is the first report for utilizing the active metabolites of seaweed for fabrication of tetrametallic nanocomposite and integration in agricultural and/or biomedical applications.

### Characterization of the TSCMZ nanocomposite

#### FT-IR analysis

**Fig. 2:**
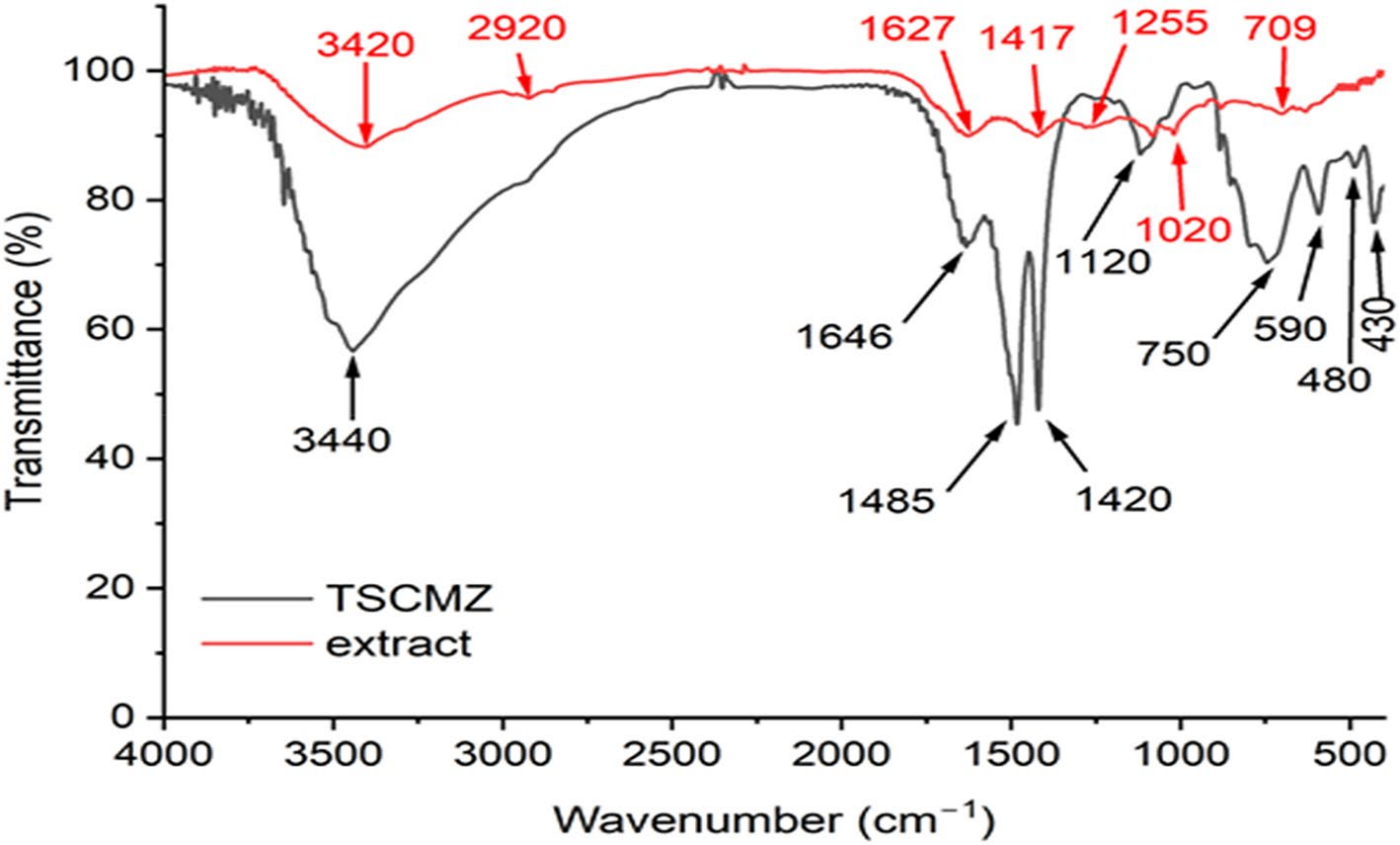
FT-IR of algal extract (red line) and biogenic nanocomposite (black line) display several absorption peaks at various wavenumbers.

In the present investigation, the brown macroalga *Sargassum latifolium* was employed for the first time as an eco-friendly platform for the biosynthesis of a tetrametallic nanocomposite. This green fabrication process is primarily due to the diverse metabolites in the algal extract, which have dual functions, reduction and capping, during nanocomposite formation. Functional groups associated with these biomolecules were determined by FT-IR, as illustrated in Fig. 2. The spectral profile of the algal aqueous extract exhibited multiple characteristic absorption bands at 3420, 1627, 1417, 1255, 1020, and 709 cm^−1^. These bands correspond to various bioactive constituents, including carbohydrates, primary and secondary amines, proteins, and amino acids, which reduce the metal precursors and subsequent stabilization of the formed nanocomposite (Table 2). Following tetrametallic nanocomposite synthesis, noticeable shifts in these absorption bands toward higher or lower wavenumbers were observed. Moreover, additional peaks appeared during nanocomposite formation, as summarized in Table 2. The FT-IR pattern of the fabricated nanocomposite displayed prominent absorption bands at 3440, 1646, 1485, 1420, and 1120 cm^−1^, along with several peaks within 400–900 cm^−1^ wavenumbers (Fig. 2; Table 2).

**Table 2:** Various band explanations observed in the FT-IR pattern of the algal extract and the fabricated nanocomposite.

Algal extract	TSCMZ nanocomposite	Vibration and references
3420 cm^–1^	3440 cm^–1^	Stretching of N–H for primary amines or stretching O–H of alcohol (intermolecular bonded)^31,32^
2920 cm^–1^	–	Medium vibration peak for stretching C-H of alkene^33^
1627 cm^–1^	1646 cm^–1^	C=O of polysaccharide, C=N stretching of amides, or overlapped C=O with N–H bending of amines^34^
1417 cm^–1^	1485 cm^–1^ and 1420 cm^–1^	stretching C═O of carboxylic salts, Stretching N–O of Nitro compounds, and CO_2_ or CO_3_^2−^ adsorption on the surface of nanocomposite^3^
1255 cm^–1^ and 1020 cm^–1^	1120 cm^–1^	Stretching S=O of sulfone, or stretching C-O, stretching C–N, or bending C–H of primary amines^35,36^
709 cm^–1^	750 cm^–1^	Bending C-H of alkenes^37^
–	590 cm^–1^, 480 cm^–1^, and 430 cm^–1^	Cu–O or Mg–O, or Zn–O overlapped with C–S from algal extract^28,38^

#### Morphological analysis

**Fig. 3:**
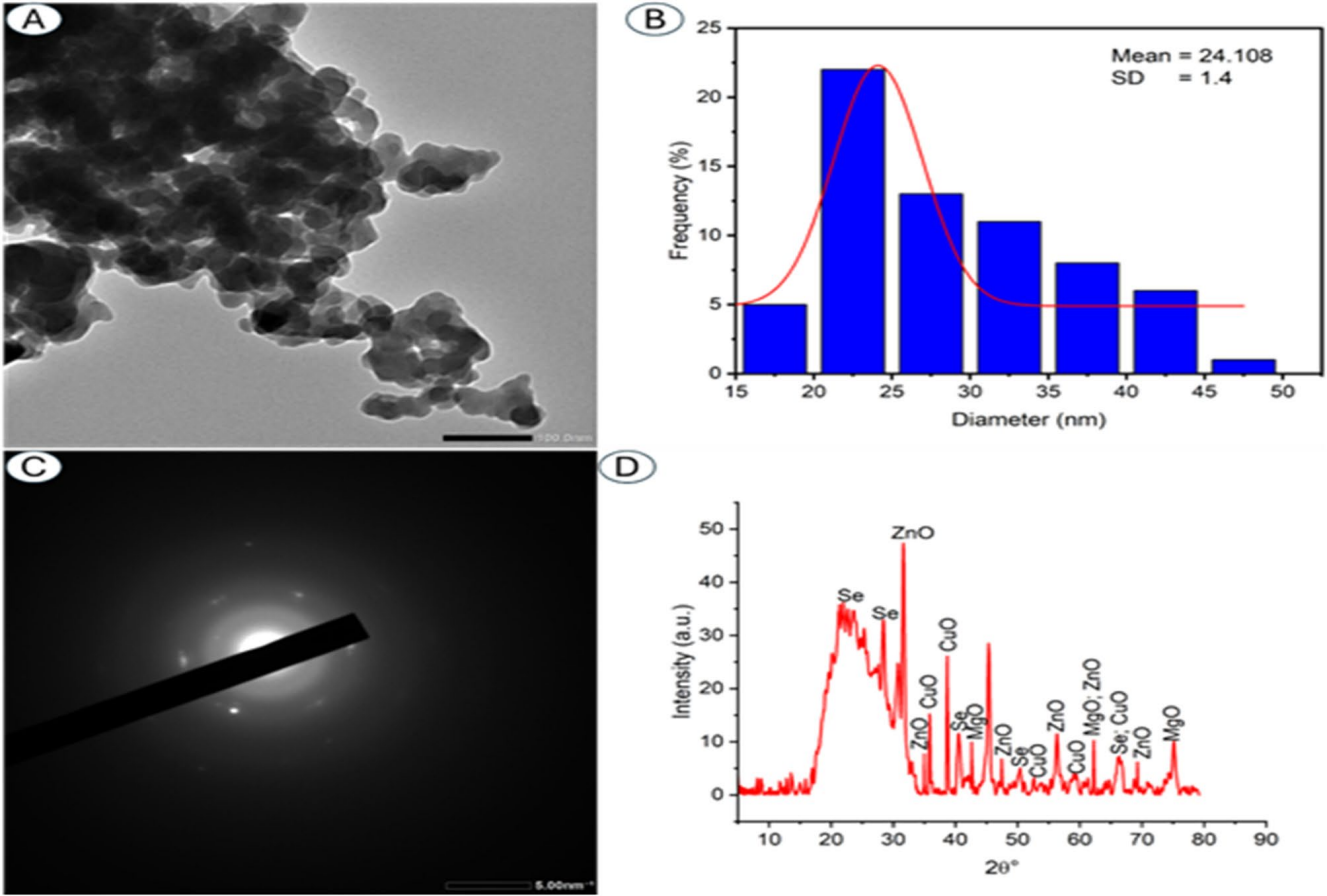
Characterization of the biogenic tetrametallic Se/CuO/MgO/ZnO nanocomposite using TEM (**A**) to examine the shape and size, size distribution (**B**), SAED (**C**), and XRD (**D**).

Nanoparticle characteristics such as particle size, morphology, and degree of aggregation are key determinants influencing their applicability across various fields^39^. Accordingly, TEM analysis of the biosynthesized nanocomposite revealed predominantly spherical shape with sizes between 15 and 45 *nm* (Fig. 3A). Moreover, the calculated average size of fabricated nanocomposite was 24.1 ± 1.4* nm* (Fig. 3B). Nevertheless, the TEM micrographs also indicated partial aggregation among the synthesized nanostructures. In a similar context, Kang et al. successfully synthesized spherical multimetallic PtPdRhRuCu nanoparticles with a mesoporous nanostructure and 23 *nm* as an average size^40^. Likewise, a pyramidal-shaped trimetallic Zn-Se-CuO nanocomposite with 26 *nm* average size was previously fabricated by *Aspergillus niger*^41^.

Selected area electron diffraction (SAED) analysis, performed in conjunction with TEM, is a powerful technique for elucidating nanoparticle crystallinity. The SAED pattern of the biogenic tetrametallic nanocomposite exhibited bright concentric rings, confirming its polycrystalline nature (Fig. 3C). The presence of discrete bright spots superimposed on these rings suggests high crystallinity and may indicate the existence of single crystalline domains embedded within a polycrystalline matrix^42^. Based on SAED patterns, MgO displayed a cubic structure characterized by sharp rings which confirmed corresponding face-centered cubic (FCC) lattice formation, while CuO exhibited a monoclinic crystal structure with well-defined diffraction features. Furthermore, ZnO was identified as having a hexagonal wurtzite structure, whereas crystalline selenium appeared, as evidenced by distinct spots or broader diffraction rings. These observations were further validated by XRD analysis.

As previously reported, nanoparticle size and morphology are among the most critical parameters governing nanocomposite activity. The ratio between NPs-surface area and volume (SA/V) explains the enhancement in activity observed by decreasing particle size. In biological systems, smaller NPs offer a large SA, enabling improved reaction with cellular components. Moreover, the increased density of active surface sites resulting from a high SA/V ratio enhances catalytic efficiency. The NPs’ size and shape strongly influence their interaction with microbial cells and their biological activity^43^. The macroalgae are characterized by their efficacy for generating smaller NPs sizes compared to microalgae due to its high secondary metabolites production. For example, *Chlorella vulgaris* was used for production of ZnO-NPs with high sizes of 150 *nm*, which may be effect on the activities^44^. Torres et al.^45^ reported that plant-mediated biogenic selenium nanoparticles exhibited superior antioxidant activity at smaller particle sizes compared with larger counterparts. Additionally, nanoparticle morphology significantly affects biological performance; cubic selenium nanoparticles demonstrated strong free-radical scavenging potential, whereas spherical particles showed enhanced antimicrobial activity^46^. Treatment of pathogenic G + bacteria (*Bacillus subtilis* and *Staphylococcus aureus)* and G- bacteria (*Pseudomonas aeruginosa* and *E. coli)* with 20 *nm* CuO nanoparticles resulted in higher inhibition percentages compared with larger particles (21, 25, and 27 *nm*)^47^. Similarly, *B. subtilis* treated with 35 *nm* MgO nanoparticles exhibited 96.1% inhibition, exceeding that observed for 47 *nm* (94.5%) and 2145 *nm* (75.7%) particles^48^. The toxicity of ZnO nanoparticles against *Raphidocelis subcapitata* was shown to depend on both size and shape, following the trend: spherical > rod-shaped and 20 *nm* > 40* nm* > 100* nm* > 500 *nm*^49^. The relatively small particle size achieved in the present study suggests promising activity across multiple applications, as discussed below.

#### Crystalline nature analysis

Biogenic tetrametallic nanocomposite-XRD analysis was performed over a 2θ range of 5–80° (Fig. 3D). The presence of well-defined Bragg reflections confirmed nanocomposite crystalline nature and verified the coexistence of selenium, CuO, MgO, and ZnO phases. Sharp diffraction peaks further supported high crystallinity structure. Selenium crystallinity was identified using JCPDS file No. 06-0362^8^, with characteristic diffraction 2θ peaks at 23.1° and 29.9°, matching the planes (100) and (101), respectively. Additional selenium-related peaks were observed at 40.2° (110), 50.09° (112), and 66.1° (210)^50^. The monoclinic structure of CuO was evidenced by diffraction 2θ peaks at 35.6° and 38.5°, assigned to the planes of (002) and (111), respectively^51^. Further CuO-related reflections at 53.6°, 58.4°, and 66.4° corresponded to the (020), (202), and (310) planes, in agreement with JCPDS file No. 80-1916^52^. As previously reported^53^, the presence of diffraction peaks within 2θ values of 35–39° confirms the reduction of copper precursors to nanoscale CuO within the composite. Diffraction peaks observed at 42.6°, 62.2°, and 75.4° corresponded to planes (200), (220), and (311) of polycrystalline cubic MgO (JCPDS file No. 87-0653)^54^. In addition, strong diffraction peaks at 31.7° (100), 43.4° (002), and 47.2° (102), confirmed the hexagonal ZnO wurtzite structure, consistent with JCPDS file No. 36-1451^55^. The XRD results were in strong agreement with SAED findings. Notably, peak overlap around 2θ values of approximately 62° and 66° suggests possible interaction or partial alloying among the different metallic components within the nanocomposite^56^. Minor additional peaks may be attributed to scattering effects arising from the algal capping layer coating the nanocomposite surface.

#### Elemental composition analysis

**Fig. 4:**
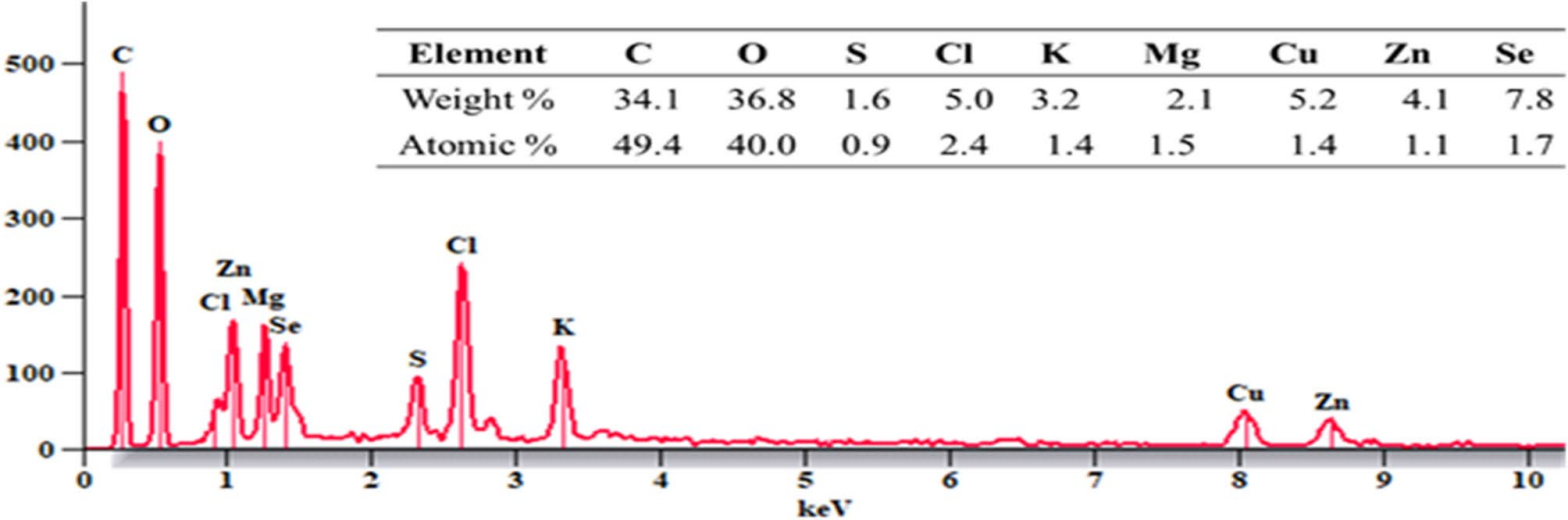
EDX analysis for tetrametallic nanocomposite showed qualitative and quantitative elemental composition.

The elemental ions of the biogenic TSCMZ were examined by EDX analysis (Fig. 4). The EDX spectrum confirmed that selenium, copper, magnesium, and zinc constituted the principal elements of the synthesized material. The oxygen signal appeared at a binding energy of 0.5 keV, while characteristic peaks for Se, Cu, Mg, and Zn were detected at approximately 1.3, 8.1, 1.2, and 1.1/8.8 keV, respectively (Fig. 4). Comparable results were previously reported for a trimetallic Se-ZnO-CuO nanocomposite, which exhibited prominent peaks corresponding to Se, Zn, Cu, and oxygen^57^. Quantitative EDX analysis revealed weight percentages of 36.8% (O), 7.8% (Se), 5.2% (Cu), 2.1% (Mg), and 4.1% (Zn), whereas atomic percentages were 40%, 1.7%, 1.4%, 1.5%, and 1.1%, respectively (Fig. 4). These findings indicate that selenium represented the most abundant metallic component following copper, zinc, and magnesium. The relatively high oxygen content may be attributed to the CuO, MgO, and ZnO phases formation or to O-adsorption from the surrounding environment onto the nanocomposite surface^58^. The detection of additional elements such as carbon, sulfur, chlorine, and potassium at lower concentrations was consistent with XRD observations and likely resulted from the algal biomolecules, which acting as capping agents for NPs-surface^37,49^.

### Effect of TSCMZ nanocomposite on *Phaseolus vulgaris* under salinity stress

#### Growth parameters

Salinity is considered the most critical abiotic constraints that severely limit *Phaseolus vulgaris* L. (common bean) growth and productivity. Therefore, in the present investigation, a tetrametallic nanocomposite composed of Se, CuO, MgO, and ZnO (TSCMZ) was applied for the first time to improve *P. vulgaris*-growth and yield under salinity compared with unstressed control plants.

Data clearly shows that salinity caused noteworthy decline in all measured growth traits, including fresh and dry weights and lengths of shoot and root, as compared with control (salinity absence) (Table 3). In contrast, TSCMZ-foliar application at concentrations of 50, 100, and 200 ppm significantly improved all growth parameters in salt-stressed groups.

**Table 3:** Effects of TSCMZ nanocomposite on morphological parameters of *Phaseolus vulgaris* plant under salinity stress.

Treatments		Shoot lengths (cm)	Root lengths (cm)	Fresh weight of shoot (g)	Dry weight of shoot (g)	Fresh weight of root (g)	Dry weight of root (g)
Control	0	20.47 ± 0.96c	6.43 ± 1.01b	0.85 ± 0.17abc	0.053 ± 0.021ab	0.013 ± 0.003abc	0.004 ± 0.002abc
Salinity	100 mM	14.67 ± 2.08d	5.00 ± 0.82b	0.36 ± 0.13d	0.020 ± 0.017b	0.005 ± 0.004c	0.002 ± 0.001c
TSCMZ (without salinity)	50 ppm	26.70 ± 2.56b	8.30 ± 1.83ab	1.00 ± 0.22abc	0.060 ± 0.010ab	0.014 ± 0.003ab	0.005 ± 0.001abc
	100 ppm	33.00 ± 1.00a	8.63 ± 0.76ab	1.12 ± 0.17ab	0.087 ± 0.015a	0.018 ± 0.002ab	0.007 ± 0.001ab
	200 ppm	36.93 ± 1.90a	12.07 ± 3.89a	1.22 ± 0.12a	0.090 ± 0.010a	0.020 ± 0.003a	0.006 ± 0.001a
Salinity + TSCMZ	50 ppm	16.00 ± 2.00cd	5.53 ± 0.75b	0.61 ± 0.14cd	0.030 ± 0.010b	0.010 ± 0.002bc	0.002 ± 0.001c
	100 ppm	17.40 ± 1.35cd	7.83 ± 1.14ab	0.72 ± 0.15bcd	0.053 ± 0.012ab	0.014 ± 0.004ab	0.003 ± 0.001bc
	200 ppm	18.63 ± 1.67cd	7.60 ± 1.73ab	0.8 ± 0.18abcd	0.060 ± 0.016ab	0.017 ± 0.003ab	0.004 ± 0.001bc
HSD		2.17	2.19	0.20	0.018	0.004	0.001

Different letters in the same column indicate significant differences (*P* ≤ 0.05). Honestly significant difference (HSD) detected by post hoc-Tukey’s test.

These data are reliable with previous reports indicating that exposure of common bean plants to 120 mM NaCl resulted in reductions of approximately 50% and 40% in root and shoot dry weights, respectively, compared with unstressed plants. Moreover, composite nanoparticles of potassium copper sulfate hydrate were reported to improve morphological characteristics under salinity stress^59^. Likewise, the application of Se, ZnO, CuO, and MgO nanoparticles has been shown to promote growth in faba bean, wheat, barley, and black cumin plants, respectively, in presence and absence of NaCl^60,61^.

#### Photosynthetic pigments

**Fig. 5:**
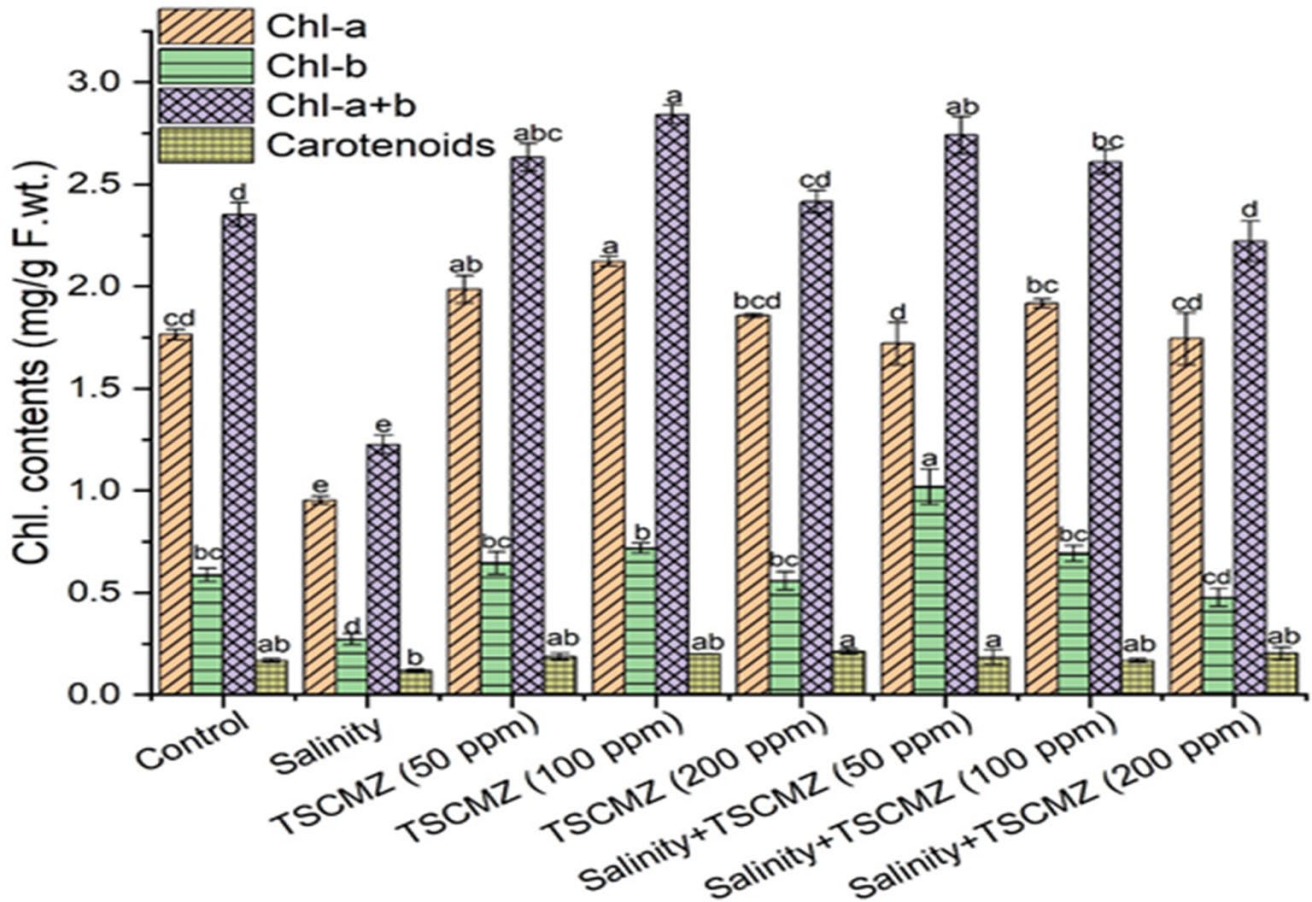
Chlorophyll (a, b and a + b) and carotenoid (mg/g fresh weight) behavior of common bean plant under salinity stress compared to control (without salinity) in presence and absence of nanocomposite. Different letters in the same bars at different groups indicate significant differences (*P* ≤ 0.05, *n* = 3).

Chlorophyll (a, b, and a + b) and carotenoids as the main photosynthetic pigments were significantly improved after TSCMZ-foliar at different concentrations (50, 100, and 200 ppm) in presence and absence of salt, compared to groups of control (absence of nanocopmposite and salt) as well as group growing under salt stress only. However, treatment with the TSCMZ nanocomposite, either alone or in combination with NaCl, significantly improve pigment contents in both stressed and unstressed common bean plants (Fig. 5).

Similarly, the faba bean-photosynthetic pigments were highly impaired upon growing under salinity^62^. This reduction has been attributed to salinity-induced damage to chloroplast ultrastructure, destabilization of pigment–protein complexes, and increased chlorophyllase activity^63^. Notably, in the current study, TSCMZ-foliar treatment at various concentrations resulted in improve of these pigment levels in both salt-treated and untreated groups. As shown, the salinity-stress plan in absence of nanocomposite treatment exhibit significant chlorophyll degradation and ionic imbalance, although all plant received equivalent amounts of Mg and K from soil fertilization. Therefore, the nanocomposite may arise the bulk Mg^2+^ supplementation, significantly improve utilization of Mg and K, and improve the Na^+^ exclusion mechanisms. The nanocomposite may facilitate the bioavailability of Mg^2+^ or modulate transporter activity, leads to significantly improved plant growth under salinity. These results highlight the effectiveness of TSCMZ in significantly alleviate salinity-induced damage and supporting plant recovery toward normal physiological status. The enhancement of nutrient uptake and overall growth by TSCMZ is likely to improve pigment biosynthesis and maintenance.

#### Metabolic contents

Plants naturally respond to stresses including salinity by accumulation of osmoprotectants, such as soluble carbohydrates, soluble proteins, and free proline, within the cytosol and other cellular compartments to mitigate stress-induced damage^64^. Herein, salinity stress significantly increased total carbohydrate, protein, and free proline contents in common bean plants in absence of any treatments.

**Fig. 6:**
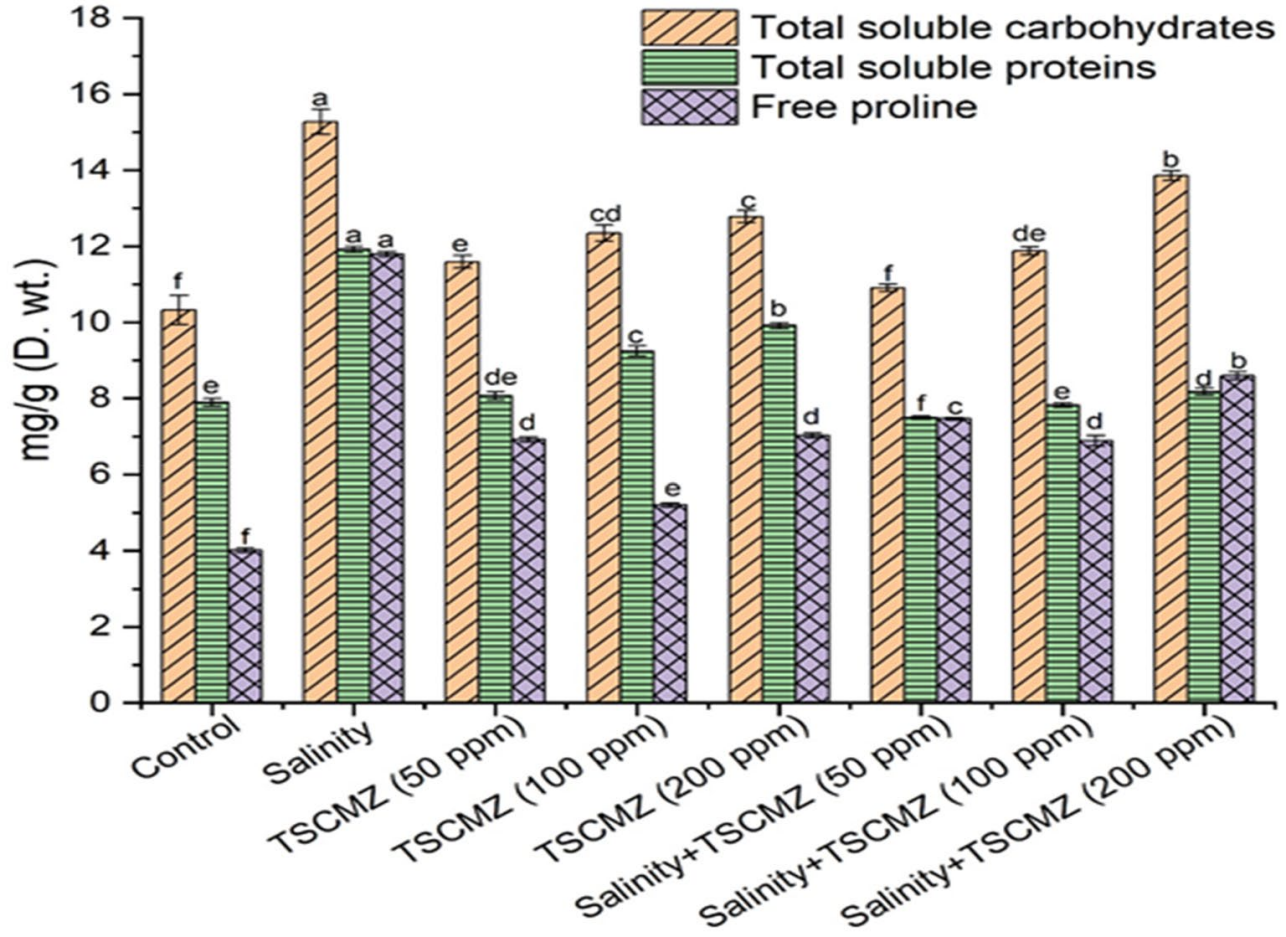
Contents of the total carbohydrates, proteins, and proline (mg/g dry weight) of *Phaseolus vulgaris* plant under salinity stress in presence and absence of TSCMZ. Significant differences (*P* ≤ 0.05) data indicated by different letters in the same bars.

In contrast, foliar TSCMZ application at all tested concentrations (50, 100, and 200 ppm) alleviated the adverse effects of salinity through a pronounced accumulation reduction of these osmoregulatory compounds (Fig. 6). Similar increases in proline, soluble proteins, and carbohydrates under salinity stress have previously been reported in rice^65^ and faba bean plants^66^. Furthermore, our findings support Ahmed et al. finding, who recorded that Zn nanoparticles enhanced total soluble carbohydrate levels in maize plants under both stressed and unstressed conditions^67^.

The decreasing of osmoprotectants upon treatment with different TSCMZ concentrations may be related to the synergistic action between nanocomposite components, which enhance plant antioxidant capacity, that reduce the damage caused by oxidative stress and the requirements of protective osmolytes. KNO_3_ was applied to all pots during soil fertilization, therefore the differences in ratios of K^+^/Na^+^ between treatments may be related to the efficacy of nanocomposite to improve K^+^ retention, reduced Na^+^ uptake, or enhance root-to-shoot K^+^ translocation in the presence of NaCl stress, rather than exogenous K^+^ supply. Therefore, TSCMZ applications improve the ions homeostasis, especially K^+^/Na^+^ balance. This ion balance decreases the osmotic imbalance which led to accumulations of carbohydrates and proline^68^.

#### Malondialdehyde contents

**Fig. 7:**
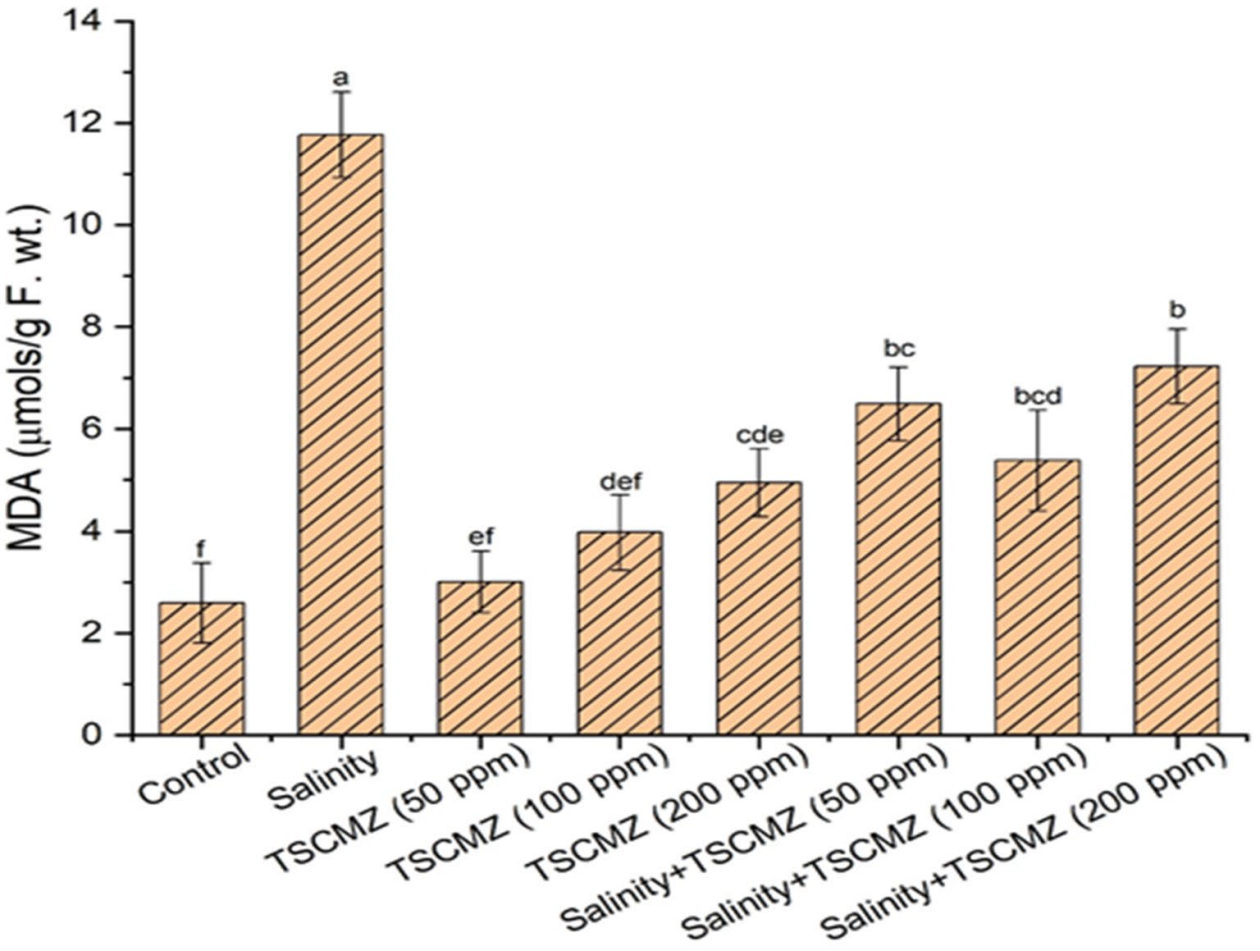
Malondialdehyde (MDA) contents (μmols/g F.wt.) of*P*. *vulgaris* plant under salinity stress in the presence and absence of TSCMZ. Significant differences (*P* < 0.05) between treatments are indicated by different letters above the bars.

Our data revealed that the presence of NaCl alone increases malondialdehyde (MDA) content in common bean leaves compared with the control. Conversely, MDA levels were markedly reduced in salt-stressed plants treated with TSCMZ (Fig. 7).

Accumulation of salt within cytoplasm causing ionic imbalance and hyperosmotic stress, resulting in overproduction of ROS, which subsequently increases membrane lipid peroxidation and elevated MDA levels^69^. In agreement with recent studies on sunflower^70^ and soybean plants^71^, common bean plants exposed solely to salinity stress exhibited significantly higher MDA concentrations. In contrast, TSCMZ application to salt-stressed plants effectively reduced lipid peroxidation and oxidative stress damage, as shown by the observed decline in MDA levels. The protective role of TSCMZ against salinity-induced oxidative stress appears to be associated with enhanced enzymatic antioxidant activity and more efficient ROS scavenging, thereby supporting plant growth and physiological stability. The presence of Se within tetrametallic nanocomposite increases the activity of glutathione peroxidase enzyme, while Cu and Zn improve the superoxide dismutase activity. The enzymatic activity because of TSCMZ treatment reduce H_2_O_2_ and MDA level compared to control (salinity in absence of treatment).

#### Enzyme activities

**Fig. 8:**
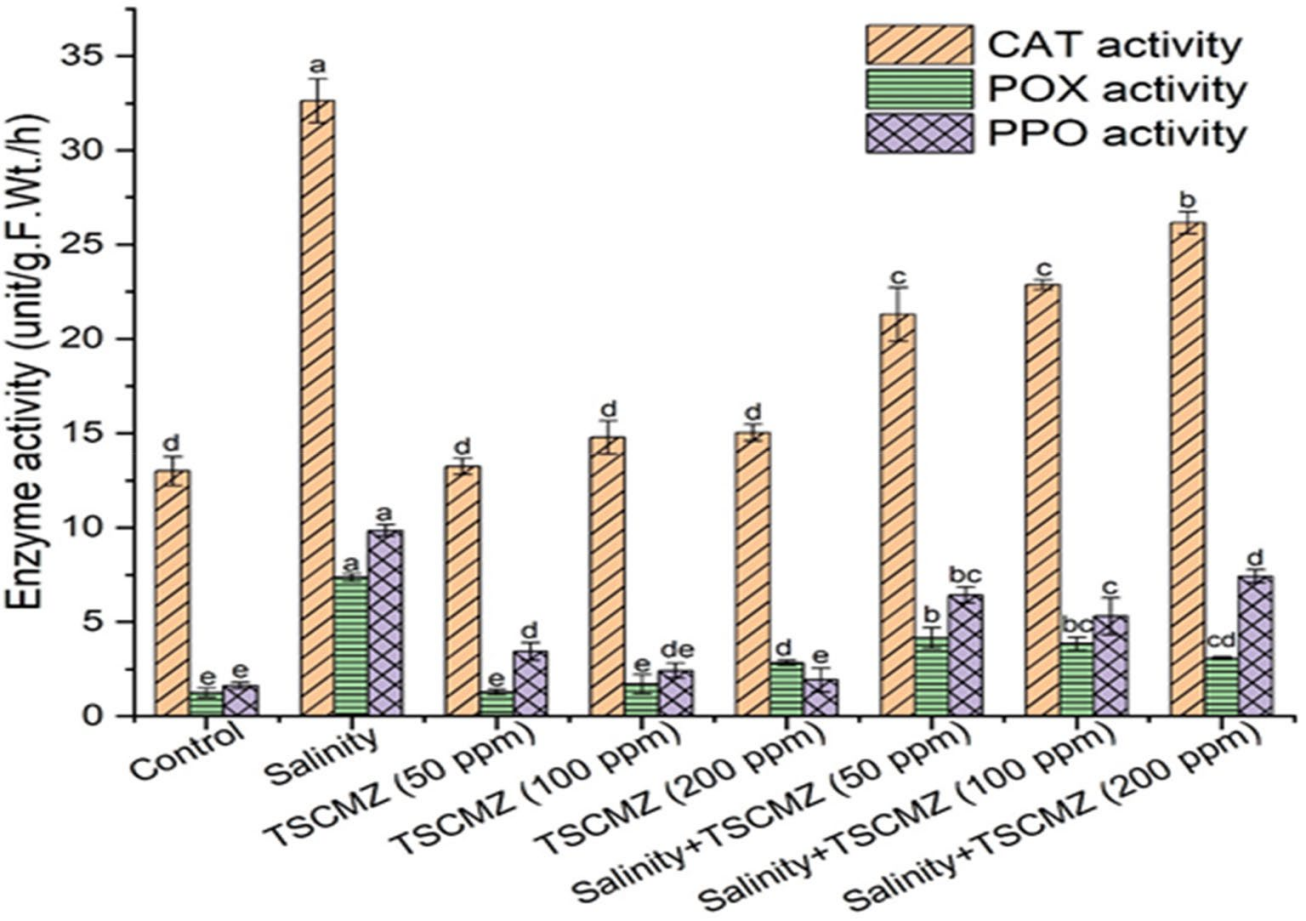
Effects of TSCMZ on different enzymes activities (unit/g.F.wt./hour) of catalase (CAT), peroxidase (POX), and polyphenol oxidase (PPO) in presence and absence of salt stress. Significant differences (*P* < 0.05) between treatments for each enzyme are indicated by different letters above the bars.

The present findings demonstrated that salinity stress significantly improve the activities of CAT, POX, and PPO enzymes in *Phaseolus vulgaris* plants compared with the control (Fig. 8). Several studies have reported that salt stress induces excessive ROS generation, which in turn activates plant defense system includes antioxidant enzymes^71,72^.

Although the precise mechanisms by which nanocomposites confer salinity tolerance remain unclear, it has been suggested that certain nanocomposites can modulate the expression of genes directly or indirectly associated with salinity stress physiology^73^. Here, the application of TSCMZ by foliar method resulted in a reduction in antioxidant enzyme activities relative to salt-stressed plants (Fig. 8). Under non-stress conditions, TSCMZ at concentrations of 200 and 50 ppm significantly enhanced POX and PPO activities, respectively.

These observations are consistent with recent reports on ZnO nanoparticles in rice under salinity stress^68^ and CuO nanoparticles in fenugreek plants^74^. Moreover, Zafar et al. reported that selenium nanoparticles upregulated key antioxidant enzymes, including POX and CAT, in wheat plants exposed to salt stress^75^. Several nanoparticles, such as ZnO, CuO, and Se, have been shown to effectively mitigate salinity oxidative stress by improving antioxidant enzymes activities, including superoxide dismutase, catalase, and peroxidase, when applied at appropriate concentrations^76^.

#### Yield trials

The results indicate that salinity stress caused a substantial reduction in yield-related traits, including the number of pods/plant, number of seeds/plant, seed weight/plant, and the weight of 100 seeds (Table 4). In addition, seed soluble carbohydrate and protein contents were significantly reduced compared with control. These results are matched by Nasiri et al., who reported a significant decline in common bean grain yield under saline conditions^77^. Similar negative impacts of salinity on yield components have been documented in wheat^78^, Pea^79^, and maize plants^80^. High generation of ROS upon salinity exposure leads to severe oxidative damage, ultimately resulting in yield reduction^81^. In contrast, nanocomposite, TSCMZ, foliar treatment alleviated the salinity negative effects and significantly improved all yield parameters in salt-stressed common bean plants. Treatment with TSCMZ at 200 ppm increased the pods number/plant (28.4%), seeds number/plant (7.73%), seed weight/plant (17.1%), weight of 100 seeds (1.66%), seed soluble carbohydrates (12.5%), and seed soluble proteins (5.29%) compared with the control under normal conditions. In the current study, the accumulation of metals in edible seeds is not detected and this is considered one of the limitations. Therefore, if the synthesized nanocomposite will be used in agricultural field, it should be detecting the metals accumulation in edible seeds before human use for toxicity assessment.

**Table 4:** Yield traits of *P. vulgaris* plant upon foliar spray of TSCMZ in presence and absence of salt stress.

Treatments		Number of pods/plants	Number of seeds/plants	Weight of seeds/plant (g)	Weight of 100 seeds (g)	Soluble carbohydrates in seeds (mg/g dry weight)	Soluble proteins in seeds (mg/g dry weight)
Control	0	4.67 ± 1.15abcd	17.33 ± 1.53a	6.93 ± 0.15c	44.48 ± 0.14b	52.79 ± 0.30d	74.81 ± 0.41b
Salinity	100 mM	1.33 ± 0.58d	10.00 ± 1.04b	5.16 ± 0.13f.	35.17 ± 0.19d	34.86 ± 0.27h	52.03 ± 0.50e
TSCMZ (without salinity)	50 ppm	5.00 ± 1.73abc	17.67 ± 1.16a	7.27 ± 0.23bc	44.90 ± 0.17a	54.59 ± 0.23c	75.32 ± 0.29b
	100 ppm	5.67 ± 1.53ab	18.00 ± 1.01a	7.44 ± 0.18b	45.03 ± 0.11a	55.30 ± 0.16b	77.27 ± 0.66a
	200 ppm	6.00 ± 1.00a	18.67 ± 0.58a	8.12 ± 0.19a	45.22 ± 0.09a	59.41 ± 0.19a	78.77 ± 0.79a
Salinity + TSCMZ	50 ppm	1.67 ± 1.16cd	11.67 ± 1.15b	5.39 ± 0.14ef	36.07 ± 0.13c	35.54 ± 0.17g	67.79 ± 0.36d
	100 ppm	2.33 ± 0.59bcd	12.00 ± 1.03b	5.88 ± 0.12d	35.14 ± 0.08c	37.46 ± 0.15f.	69.21 ± 0.35d
	200 ppm	3.33 ± 1.52abcd	12.33 ± 0.55b	5.99 ± 0.10de	36.39 ± 0.12d	39.61 ± 0.19e	71.68 ± 0.84c
HSD		1.50	1.27	0.19	0.16	0.26	0.69

Significant differences (*P* ≤ 0.05, *n* = 3) in the same species represented by different letters within a column. HSD is honestly significant difference by post hoc-Tukey’s test.

These data are consistent with recent studies reporting that selenium nanoparticles significantly enhanced yield attributes in *Oryza sativa* grown under salinity^82^. Also, Salcido-Martinez et al. concluded that foliar application of magnesium nanoparticles significantly improved yield components in green bean plants^83^.

### Antibacterial activity against multidrug resistant (MDR) bacteria

The emergence and distribution of bacterial resistance to antibiotics represent global challenges to public health. Although antibiotics remain indispensable for reducing morbidity and mortality associated with bacterial infections, their excessive and often inappropriate use has increased the resistance of bacterial strains. As a result, infections caused by MDR have become difficult to treat, hence illness prolonged which increases costs of healthcare, ultimately elevating mortality rates. Alarmingly, recent epidemiological reports indicate that antimicrobial resistance is now considered one of the mainly death worldwide, surpassing several major infectious diseases^84^.

In this context, nanotechnology-based antimicrobial strategies have attracted significant attention as promising alternatives to traditional antibiotics. The current study demonstrated that the tetrametallic TSCMZ nanocomposite synthesized *via Sargassum latifolium* exhibited strong antibacterial activity against isolated MDR strains compared to positive control (gentamicin) against some strains. The pronounced inhibitory effects observed could be related to the synergistic interactions among the four metallic components, which collectively enhance bacterial membrane disruption, intracellular ion imbalance, and reactive oxygen species (ROS) generation. Such synergism enables multimetallic nanocomposites to outperform single-metal nanoparticles, offering an efficient approach to combating MDR pathogens through multiple antibacterial mechanisms operating simultaneously. Notably, *Escherichia coli* and *Klebsiella pneumoniae*, along with *Acinetobacter baumannii*, *Staphylococcus aureus*, *Pseudomonas aeruginosa*, and *Streptococcus pneumoniae*, are widely recognized as some of the most clinically significant antimicrobial-resistant pathogens worldwide, contributing substantially to global morbidity and mortality^85^.

Here, biogenically synthesized TSCMZ *via* *Sargassum latifolium* demonstrated potent inhibition against all tested clinical isolates, with inhibition zones (IZs) in between 11.3 ± 0.6 mm to 37.3 ± 1.2 mm. The greater effect is likely because of the synergistic interactions among the four metal components, which confer higher activity than single-metal nanoparticles^86^. The negative control (DMSO) did not show any inhibition activity against tested clinical isolates.

**Fig. 9:**
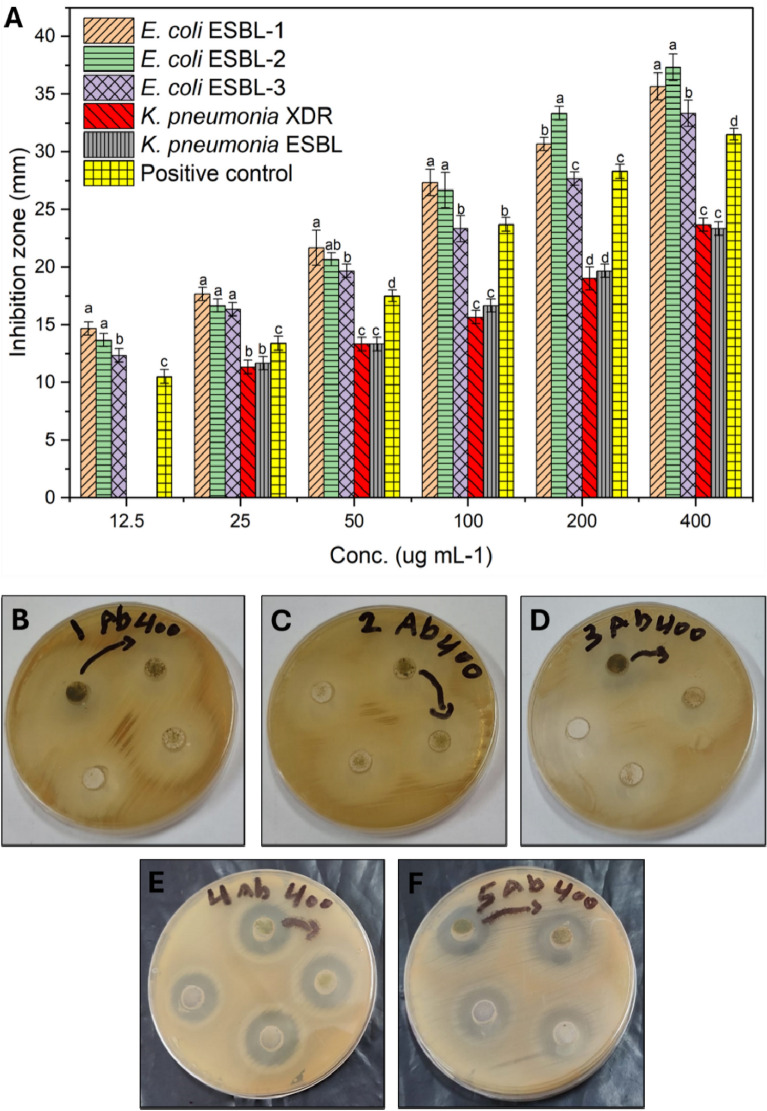
(**A**) Effect of different concentrations of green synthesized nanocomposite compared to positive control on the tested bacterial strains. Data are presented as mean ± SD of three independent experiments. Statistical analysis was performed using one-way ANOVA considering each strain–concentration combination as an independent experimental group, followed by Tukey’s multiple comparison post-hoc test (*P* < 0.05). Different letters above the bars indicate statistically significant differences among groups. (**B**–**F**) Representative agar plates showing inhibition zones for MDR clinical pathogens *E. coli*-ESBL1, *E. coli*-ESBL2, *E. coli*-ESBL3, *K. pneumoniae*-ESBL, and *K. pneumoniae*-XDR respectively.

TSCMZ was more effective against *E. coli* isolates than *K. pneumoniae*. At 400 µg mL^–1^, the IZ were 35.7 ± 1.2 mm, 37.3 ± 1.2 mm, 33.3 ± 1.1 mm, 23.7 ± 0.6 mm, and 23.3 ± 0.6 mm for *E. coli-*ESBL1, *E. coli-*ESBL2, *E. coli-*ESBL3, *K. pneumoniae-*XDR, and *K. pneumoniae-*ESBL, respectively, compared to inhibition zone of 31.5 ± 0.5 mm for positive control (gentamicin) (Fig. 9). As shown, activity of the synthesized nanocomposite against *E. coli* at high concentration is higher than the positive control, while it lower for *K. pneumoniae* strains. Comparable broad-spectrum antibacterial activity has been observed for AgCu bimetallic nanoparticles^87^, and Cu/Cr/Ni trimetallic nanocomposites, which disrupt protein structures and bacterial membranes^88^.

Our findings also revealed a dose-dependent inhibition by TSCMZ; MIC values were 12.5 µg mL^–1^ for all *E. coli* strains and 25 µg mL^–1^ for *K. pneumoniae-ESBL* and *K. pneumoniae-XDR* (Fig. 9). Similarly, the green CdO/ZnO/V_2_O₅ trimetallic nanocomposite at 10 µg mL^–1^ showed substantial antibacterial and antifungal activities toward *E. coli*, *K. pneumoniae*, *Shigella dysenteriae*, *Staphylococcus epidermidis*, *P. aeruginosa*, *A. baumannii*, *Bacillus subtilis*, *Enterococcus faecalis*, *Salmonella enterica*, *S. aureus*, and *S. pyogenes*^89^. Likewise, the tetra-metallic Fe_3_O_₄_@Cu,Mn,Ni,Co nanocomposite synthesized using rice husk displayed strong antibacterial activity against both Gram-positive and Gram-negative bacteria^90^. The antibacterial mechanisms of metallic nanocomposites may include cell wall disruption, increased membrane permeability, interference with metabolic pathways, and induction of reactive oxygen species (ROS) overlapping with NAD oxidation^91^. Light activation further enhances ROS generation, resulting in DNA and membrane damage and promoting penetration into biofilms, thereby inhibiting *P. aeruginosa* and *S. aureus*^92^. Trimetallic Ag–Cu–Li nanorods at very low concentrations (20 µg mL^–1^) have also been shown to exhibit pronounced antibacterial activity *via *ion release^93^.

The synergistic interaction of Se, CuO, ZnO, and MgO within the tetrametallic nanocomposite enhances antimicrobial activity against diverse bacteria, stabilizes the particles, and minimizes aggregation^94^. Released ions (Cu^2+^, Zn^2+^, Mg^2+^) interferes with macromolecule functions such as enzymes, proteins, and nucleic acids, causing dysfunction and promoting ROS accumulation^95^. Selenium additionally disrupts enzyme activity by replacing sulfur in thiol-containing proteins^96^. ZnO and CuO can inhibit bacterial efflux pumps associated with multidrug resistance, raising intracellular antibiotic concentrations and leading to cell death^97^. Furthermore, electrostatic interactions between positively charged nanocomposites and negatively charged bacterial membranes may damage the cytoplasmic membrane, causing leakage of cellular contents, ion imbalance, and suppression of metabolism^26,98^.

### Antioxidant activity of TSCMZ nanocomposite

Free radicals can damage and modify cellular structures, significantly impacting human health. To counteract these effects, reactive oxygen species (ROS) must be scavenged by antioxidants. The in vitro antioxidant activity of nanocomposites^99^ suggests their potential pharmacological benefits. Currently, the DPPH scavenging assay is the most widely known and commonly employed spectrophotometric for evaluating antioxidant scavenging capacity^100^. To assess the radical scavenging ability of our biogenically synthesized tetrametallic nanocomposite, ten ethanolic concentrations (1000 to 1.95 µg mL^–1^) were prepared and analyzed using the reliable DPPH assay, with reference standard of ascorbic acid.

**Fig. 10:**
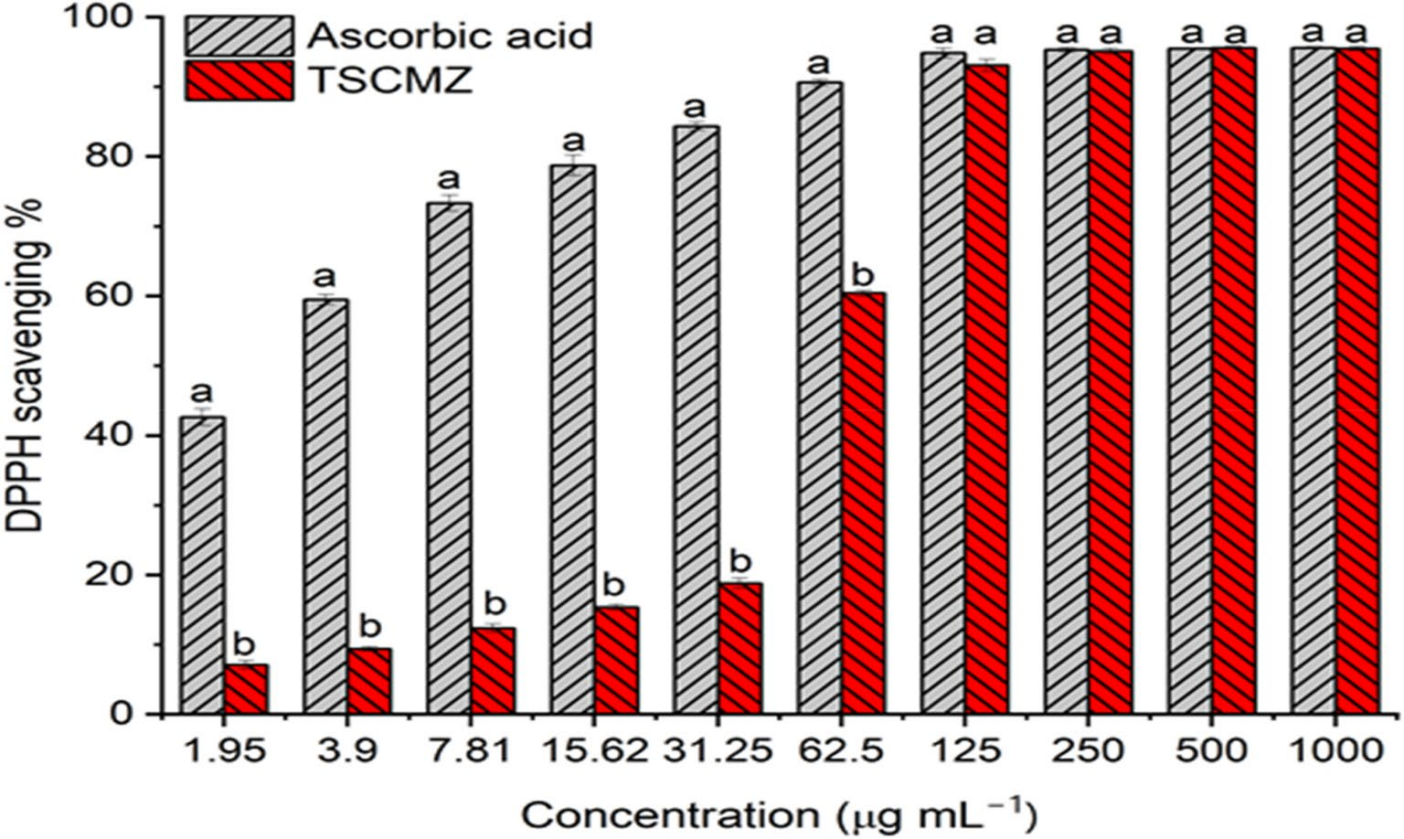
Antioxidant activity of *S. latifolium* derived TSCMZ nanocomposite at various concentrations compared to ascorbic acid (standard antioxidant).

The TSCMZ nanocomposite exhibited dose-dependent scavenging of DPPH radicals. The minimum scavenging percentages (7.1 ± 0.6%) was observed at 1.95 µg mL^–1^, whereas the maximum scavenging (95.1 ± 0.4%) occurred at 250 µg mL^–1^. For comparison, ascorbic acid showed 42.6 ± 1.2% scavenging at 1.95 µg mL^–1^ and 95.3 ± 0.3% at 250 µg mL^–1^ (Fig. 10). Furthermore, no significant differences were shown in the DPPH scavenging percentage between TSCMZ doses ≥ 125 µg mL^–1^ and ascorbic acid (Fig. 10). Owing to its multifunctional properties, including both antibacterial and anticancer effects, these results highlight the potential application of biogenic nanocomposites in areas where ascorbic acid may be unsuitable, such as environmental protection, food, and cosmetic industries. The distinctive physicochemical characteristics of each metallic component contribute to a synergistic anti-radical effect in the quaternary nanocomposite. Likewise, a Cu-Ni-Zn oxide ternary nanocomposite synthesized using *Dodonaea viscosa* aqueous leaf extract displayed superior DPPH scavenging ability compared to ascorbic acid^101^.

Phytochemical investigations have shown that *Sargassum* species contain a substantial array of bioactive substances such as anthraquinones, quinones, and coumarins. Also, compounds like flavonoids, saponins, tannins, alkaloids, triterpenes, sterols, and phenolic complexes found in this algal strain exhibit antioxidant activity. Active groups like C–C, –COOH, –NH, C=O, –CH, and –OH are present in the aqueous extract, which likely enhance the radical scavenging efficiency of the resulting tetra-metallic nanocomposite, particularly through the hydroxyl groups of phenolic compounds^34^.

The remarkable antioxidant effect of CuO/ZnO nanoparticle combinations derived from *Annona glabra* leaf extract greatly surpasses that of individual CuO or ZnO nanoparticles. The bimetallic nanoparticles exhibit synergistic DPPH scavenging because of their exceptional physicochemical attributes, including high redox potential, extensive surface area, and facilitation of electron transfer to neutralize free radicals^102^. Similarly, ZnO/MgO bimetallic nanoparticles prepared using *Citrus limonium* juice achieved over 90% DPPH scavenging at 850 µg mL^–1^^103^. This potent antioxidant activity was attributed to enhanced surface-induced electron–hole pair generation, which promotes the formation of hydrogen and hydroxyl radicals from water, thereby improving redox reactions.

## Conclusion

The present study confirms that algal-mediated green synthesis of a tetrametallic Se/CuO/MgO/ZnO (TSCMZ) nanocomposite using the aqueous extract of the brown macroalga *Sargassum latifolium* represents an economical, environmentally friendly, and sustainable strategy for nanomaterial production. Comprehensive physicochemical characterization using FT-IR, TEM, SAED, EDX, and XRD analyses verified the successful formation of a well-defined, polycrystalline nanocomposite with predominantly spherical morphology and an average particle size of approximately 24 *nm*. Elemental analysis further confirmed the homogeneous incorporation of selenium, copper, magnesium, zinc, and oxygen within the nanocomposite matrix. Foliar application of the biosynthesized TSCMZ nanocomposite significantly improved growth performance and yield characteristics of common bean (*Phaseolus vulgaris* L.) under salinity stress compared with untreated salt-stressed plants, highlighting its capacity to partial alleviate salinity-induced physiological limitations and improve plant tolerance under adverse environmental conditions.. Beyond its agricultural benefits, the TSCMZ nanocomposite exhibited strong antibacterial activity against multidrug-resistant bacterial strains, as reflected by low minimum inhibitory concentration values. Furthermore, the nanocomposite demonstrated potent antioxidant activity, efficiently scavenging DPPH radicals at higher concentrations (125–1000 µg mL^−1^), with effectiveness comparable to ascorbic acid. Overall, these findings emphasize that the *Sargassum*-derived tetrametallic nanocomposites is an effective proof-of-concept treatment to increase the crop tolerance to salinity stress, as well as exhibiting antimicrobial and antioxidant activities. However, the current study has some limitations that should be addressed before practical application of the obtained findings. Among these limitations, some important agricultural parameters were not evaluated, such as nodulation parameters, rhizosphere/phyllosphere microbial communities, soil microbial biomass, and the possible effects of foliar runoff on soil microbiota. In addition, the accumulation of metals in edible seeds should be assessed before human consumption to ensure safety. Furthermore, comparative studies between nanoscale metal structures and their corresponding non-nano metal forms at the same equimolar concentration are still required.

## Data Availability

This manuscript does not report data generation or analysis. All data supporting the findings of this study are available within the paper.
